# The interactive effect of diabetes and central obesity on stroke: a prospective cohort study of inner Mongolians

**DOI:** 10.1186/s12883-015-0328-y

**Published:** 2015-04-28

**Authors:** Jennifer Olofindayo, Hao Peng, Yan Liu, Hongmei Li, Mingzhi Zhang, Aili Wang, Yonghong Zhang

**Affiliations:** Department of Epidemiology, School of Public Health, Medical College of Soochow University, 199 Ren-ai Road Industrial Park District, Suzhou, China; Department of Epidemiology, Tulane University School of Public Health and Tropical Medicine, New Orleans, LA USA; Jiangsu Key Laboratory of Preventive and Translational Medicine for Geriatric Diseases, School of Public Health, Soochow University, Suzhou, China

**Keywords:** Central obesity, Diabetes, Stroke, Interaction, Mongolians

## Abstract

**Background:**

The relationship between central obesity and stroke is inconsistent in diabetic and non-diabetic populations. This indicates an interaction between diabetes and central obesity on stroke risk. The present study aimed to examine the interaction in a cohort of Inner Mongolians.

**Methods:**

In this prospective cohort study, we assessed the interaction between diabetes and central obesity on stroke incidence between June 2003 and July 2012. At baseline, 2,589 adults were recruited and examined from Inner Mongolia, China. Participants were categorized into four subgroups according to presence of diabetes and/or central obesity. Both additive and multiplicative interactions were evaluated using Cox proportional-hazard models.

**Results:**

121 stroke events were recorded during the follow-up period. The cumulative incidence of stroke was highest for participants with both diabetes and central obesity (log-rank test, *P* = 0.042). The multivariable-adjusted risk for stroke was significantly higher in participants with both conditions (HR = 3.02, 95% CI 1.24-7.33, *P =* 0.015) compared to those with neither diabetes nor central obesity. Attributable proportion due to the interaction between diabetes and central obesity was 0.571 (95% CI 0.017-1.125). The multiplicative interactive effect between diabetes and central obesity on stroke was also statistically significant (HR = 2.67, 95% CI 1.14-6.26, *P* = 0.024).

**Conclusions:**

The participants who were both diabetic and centrally obese had significantly higher risk for incident stroke than the combination of individuals who individually had either condition among Mongolian population. This study suggests that central obesity and diabetes act synergistically to increase the risk of stroke.

## Background

Stroke is most commonly known as the second leading cause of death worldwide and the first leading cause of adult disability [1,2]. Diabetes is a clear risk factor for stroke [1-3], although intensive management of diabetes has not shown significant reduction of risk for stroke [4]. A high waist circumference, which is predictive of central obesity, has been found to be a strong risk factor for diabetes [5]. Although recent studies identify central obesity as one of the most powerful predictors of stroke in patients with diabetes [6], numerous guideline statements for stroke prevention categorize obesity as a “less documented or potentially modifiable risk factor for stroke” [7,8]. Limited studies have examined the relationship between central obesity as defined by waist circumference and stroke. It has been reported that central obesity increases an individual’s risk of developing diabetes [9,10], and diabetes has been associated with increased risk of stroke [11], central obesity may have biological interactive effect with diabetes on incident stroke. However, the interaction of central obesity and diabetes on the risk of stroke has not been well documented. The aim of this study was to examine the interaction of diabetes and central obesity on incident stroke among Mongolians, in Inner Mongolia, China.

## Methods

### Study population

All participants were at least 20 years of age and were recruited from 32 villages in two neighboring townships located in the counties of Kezuohou Banner and Naiman Banner in Inner Mongolia. Most of the residents of these townships are Mongolians who have lived there for many generations and maintain a traditional diet and lifestyle. At baseline, in 2003, this study included 2,589 individuals (1064 males and 1525 females) all without previous cardiovascular disease (CVD) including stroke. The subjects were selected from the 32 villages. The selection criteria were to meet all of the followings: (1) age: ≥ 20 years, (2) ethnicity: Mongolian. There were a total of 3,475 eligible residents in the study fields. The exclusion criteria were to meet one of the followings: (1) self-reported history of CVD, stroke, or tumors, (2) taking antihypertensive medications, (3) being pregnant. The 886 people who refused to participate or met the exclusion criteria were excluded. In 2012, 2,583 individuals (99.8%) were successfully contacted to provide comprehensive health information. All participants provided written informed consent. The present analysis is based on the baseline and follow-up examinations. The ethics committee at Soochow University in China approved this study.

### Data collection

Participants underwent a thorough physical examination at baseline and the last year of follow-up where anthropometric information, blood pressure measurements, and blood samples were obtained. Data on demographic information, lifestyle risk factors, family history of CVD, and personal medical history were gathered from standard questionnaires written in Chinese and administered by trained staff. Smoking and drinking were two lifestyle risk factors that were pertinent to this study. An individual was classified as current cigarette smoker if they smoked at least 1 cigarette per day for 1 year or more leading up to the start of the study. An individual was classified as current drinker if they consumed any type of alcoholic beverage at least once per week during the last three years.

A physical examination was then administered. After the participants had been resting for 5 minutes, they remained seated and three consecutive blood pressure measurements (3 minutes between each) were made with a standard mercury sphygmomanometer and an appropriately sized cuff [12]. The first and fifth Korotkoff sounds were recorded as systolic and diastolic blood pressure, respectively. The mean of the 3 physician-obtained easurements constituted the reported blood pressure used in further analyses. In our study, hypertension was defined as systolic blood pressure (SBP) of at least 140 mmHg and/or diastolic blood pressure (DBP) of at least 90 mmHg. Body weight and height were measured using standard methods, and body mass index (BMI) was calculated as weight in kilograms divided by the square of the height in meters (kg/m^2^). Waist circumference (WC) was measured 1 cm above the umbilicus. Women with a WC greater than 80 cm and men with a WC greater than 85 cm were classified as centrally obese [13].

Blood samples were obtained by venipuncture in the morning after a requested overnight fast (at least 8 hours). All plasma and serum samples were frozen at −80°C until laboratory testing. Fasting plasma glucose (FPG) was measured using a modified hexokinase enzymatic method [14]. Diabetes was defined as one of the following: (1) FPG ≥ 7 mmol/L (2) self-reported history of diabetes or (3) current use of either insulin or oral diabetes medication [15]. Serum total cholesterol (TC), high-density lipoprotein cholesterol (HDL-C), and triglycerides (TG) were assessed enzymatically using commercial reagents [14]. Low-density lipoprotein cholesterol (LDL-C) concentration was calculated by means of the Friedewald equation for participants who had less than 400 mg/dL TG [16].

### Outcome assessment

Participant follow-up was executed between June 2003 and July 2012. In this study, stroke as defined by both ischemic and hemorrhagic stroke was the event of interest. Stroke was defined as a sudden critical onset of neurological symptoms lasting at least 24 hours [17]. Participants were also diagnosed by cranial computed tomography or magnetic resonance imaging (MRI). Trained staff interviewed either the participants or their relatives, if participants were dead or unable to communicate, every two years to find new stroke cases. When a new case was found during follow-up, the staff reviewed the hospital records and completed a standard event form. An end point review committee made the final decisions regarding a participants’ stroke diagnosis.

### Statistical analysis

Baseline characteristics were analyzed for the total population. In the results continuous variables that were not normally distributed were expressed as median and interquartile range, normally distributed continuous variables were expressed as mean ± standard deviation, and binary variables were expressed as frequency and percent.

Participants were then divided into four categories: individuals having neither risk factor, only diabetes, only central obesity, or both risk factors. The distributions of other conventional risk factors were compared across the four subgroups mentioned. Normally distributed continuous variables were assessed by ANOVA, continuous variables with a skewed distribution were assessed by Wilcoxon rank-sum test, and categorical variables were assessed by a Chi-square test. Univariate and multivariate Cox proportional hazard models were utilized to calculate the hazard ratios (HRs) and 95% confidence intervals (95% CI) for each category compared to individuals with neither risk factor. A multiplicative interaction term of diabetes and central obesity was set in the models to test the presence of an effect. In the multivariate models, some important confounders at baseline such as age, gender, TC, TG, family history of CVD, smoking, drinking, and hypertension were all included as covariates. The biological additive interaction between diabetes and central obesity on stroke was evaluated by three indexes: relative excess risk because of interaction (RERI), attributable proportion because of interaction (AP), and synergy index (S) [18]. If there was no biological interaction the 95% CI of RERI and AP would include 0 and the 95% CI of S would contain 1. Finally, the yearly cumulative incidence of stroke among the four subgroups was estimated using Kaplan-Meier survival curves and compared using the log-rank test. A two-tailed *P* value < 0.05 was considered statistically significant. All analyses were completed using SAS 9.1 software.

## Results

### Baseline characteristics

Among the 2589 individuals included in the study at baseline, 6 were lost to follow-up and 22 were excluded from further analyses due to missing WC or FPG. A total of 2561 participants were included in the final analyses. The study sample was comprised of 1513 women and 1048 men, with a combined mean age of 46.5 years. After being followed for a mean of 9.2 years and contributing approximately 23,292 person-years, 121 stroke events (75 ischemic, 44 hemorrhagic, and 2 unknown subtypes) were observed. The cumulative incidence of stroke was 4.78%. With only 6 participants lost, the follow-up rate for this study was 99.8%. Table 1 lists the baseline characteristics for all the participants. The mean FPG measurement was 4.99 mmol/L and 3.67% of the participants were diabetic at baseline. Average WC and BMI were 80.78 cm and 22.26 kg/m^2^, respectively. 37.29% of the participants were hypertensive at baseline with the average blood pressure being 129.7/84.5 mmHg. Average TC, TG, LDL-C, and HDL-C were 3.74, 1.26, 2.31, 1.17 mmol/L, respectively. There were 1137 (44.4%) cigarette smokers, 855 (33.39%) alcohol consumers, and 334 (13.04%) participants who reported having family history of CVD.

**Table 1:** **Baseline characteristics**

**Variables**	**Mean/N(%)**	**SD/95% CI**
No. of participants	2561	
Age, years	46.49	12.38
Male, n(%)	1048(40.92)	39.01 - 42.85
TC, mmol/L	3.74	1.13
TG, mmol/L	1.26	1.29
LDL-C, mmol/L	2.31	1.03
HDL-C, mmol/L	1.17	0.33
FPG, mmol/L	4.99	1.21
BMI, kg/m^2^	22.26	3.46
WC, cm	80.78	9.57
SBP, mmHg	129.7	24.6
DBP, mmHg	84.5	12.9
Family history of CVD, n(%)	334(13.04)	11.76 - 14.41
Current smoker, n(%)	1137(44.4)	42.46 - 46.35
Current drinker, n(%)	855(33.39)	31.56 - 35.25
Hypertension, n(%)	955(37.29)	35.41 - 39.2
Diabetes, n(%)	94(3.67)	2.98 - 4.47
Dyslipidemia, n(%)	1083(42.29)	40.37 - 44.23
Central obesity, n(%)	1058(41.31)	39.4 - 43.25

TC, total cholesterol; TG, triglycerides; LDL-C, low density lipoprotein cholesterol; HDL-C, high density lipoprotein cholesterol; FPG, fasting plasma glucose; BMI, body mass index; WC, waist circumference; SBP, systolic blood pressure; DBP, diastolic blood pressure; CVD, cardiovascular disease. SD, standard deviation.

Table 2 presents the baseline characteristics of participants by the 4 study subgroups: participants with neither diabetes nor central obesity, participants with either diabetes or central obesity, and participants with both diabetes and central obesity. Conventional stroke risk factors such as age, sex, BMI, blood pressure, blood lipids, FPG, smoking, and family history of CVD were significantly different among the 4 subgroups. Central obese participants with or without diabetes tended to be smokers and have higher TC, TG, LDL-C, BMI, SBP, and DBP than those with normal WC. Participants with both diabetes and central obesity were more likely to be older, current smokers, and have higher TC, TG, LDL-C, BMI, SBP, DBP, and family history of CVD compared with those with neither diabetes nor central obesity.

**Table 2:** **Distribution of the potential confounders among participants with or without diabetes and central obesity**

**Variables**	**No diabetes**	**No diabetes**	**Diabetes**	**Diabetes**	***P*** **value**
	**No central obesity**	**Central obesity**	**No central obesity**	**Central obesity**	
No. of participants	1463	1004	40	54	
Age, years	44.73±12.54	48.61±11.63^*^	50.13±15.25^*^	52.02±11.11^*^	<0.001
Male, n(%)	693(47.37)	314(31.27)^*^	21(52.5)^†^	20(37.04)^*‡^	<0.001
TC, mmol/L	3.42(2.83-4.08)	3.87(3.24-4.68)^*^	3.54(2.54-4.6)^†^	4.42(3.33-5.25)^*‡^	<0.001
TG, mmol/L	0.83(0.6-1.21)	1.12(0.76-1.66)^*^	0.97(0.79-1.4)^*^	1.78(1.31-2.87)^*#‡^	<0.001
LDL-C, mmol/L	2(1.5-2.58)	2.41(1.85-3.17)^*^	2.32(1.3-3.29)	2.59(1.69-3.69)^*‡^	<0.001
HDL-C, mmol/L	1.17(0.98-1.4)	1.11(0.93-1.32)	1.09(0.96-1.27)	1.05(0.94-1.35)	0.053
BMI, kg/m^2^	20.3±2.02	24.99±3.15^*^	20.03±1.81^†^	25.98±3.3^*†‡^	<0.001
SBP, mmHg	125.89±23.22	134.2±25.09	130.43±19.84	146.69±33.42^*†‡^	<0.001
DBP, mmHg	82.29±12.13	87.42±13.18^*^	84.13±10.14	90.5±15.33^*‡^	<0.001
Hypertension, n(%)	427(29.19)	475(47.31)^*^	20(50.00)^*^	33(61.11)^*†‡^	<0.001
Family history of CVD, n(%)	170(11.62)	138(13.75)	14(35)^*†^	12(22.22)^*†‡^	<0.001
Current smoker, n(%)	724(49.49)	372(37.05)^*^	21(52.5)^†^	20(37.04)^*‡^	<0.001
Current drinker, n(%)	517(35.34)	308(30.68)	13(32.5)	17(31.48)	0.116

^*^Compared to participants with no diabetes and no central obesity, *P* <0.05; ^†^Compared to participants with no diabetes and central obesity, *P* <0.05; ^‡^Compared to participants with diabetes but no central obesity, *P* <0.05.

### Interactive effect of diabetes and central obesity on stroke

**Figure 1:**
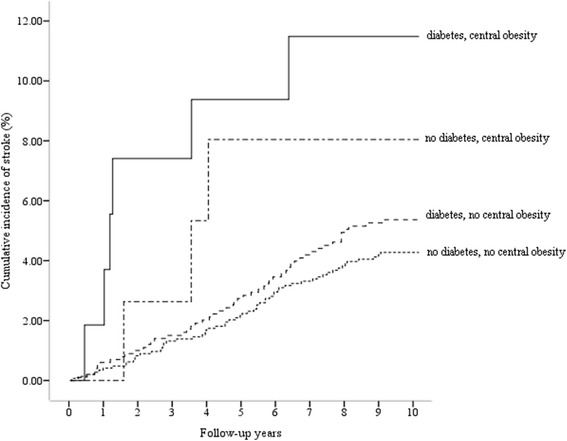
Cumulative incidence of stroke by diabetes and/or central obesity risk categories. For comparison of cumulative incidence distribution among categories, the log-rank test was used (log-rank test, *X* ^2^ = 8.209, *P* = 0.042).

Figure 1 depicts the cumulative incidence of stroke by the 4 risk categories for each year of follow-up. The cumulative incidence of stroke for participants without diabetes or central obesity (4.10%) was significantly lower than that for those with both diabetes and central obesity (11.1%) (log-rank test, *P* = 0.042). The participants with neither diabetes nor central obesity were used as the reference group to calculate subsequent measures of association. Although there was an observed increase in risk of incident stroke among participants with either diabetes or central obesity, the increase was not statistically significant when compared to those with neither risk factors (Table 3). In contrast, participants with both diabetes and central obesity had a significantly increased risk for incident stroke (HR = 2.94, *P* = 0.012). The multiplicative effect between diabetes and central obesity on stroke was also statistically significant (HR = 2.63, *P* = 0.021). After adjusting for important variables such as age, gender, TG, TC, family history of CVD, drinking, smoking, and hypertension, risk for incident stroke remained significantly increased in participants with both diabetes and central obesity, compared to those with neither (HR = 3.02, *P* = 0.015). The HR of stroke for individuals with both diabetes and central obesity was higher than the sum of the HR for individuals with only diabetes and individuals with only central obesity. Also after adjustment, the multiplicative effect between diabetes and central obesity on stroke remained significant (HR = 2.67, *P* = 0.024).

**Table 3:** **Interactive effect analysis of diabetes and central obesity on stroke**

**Categories**		**Stroke/n**	**Unadjusted**		**Adjusted**	
**Diabetes**	**Central obesity**		**HR (95% CI)**	***P*** **value**	**HR (95% CI)**	***P*** **value**
(-)	(-)	60/1463	1.00(reference)		1.00(reference)	
(-)	(+)	52/1004	1.26(0.87-1.83)	0.219	1.22(0.82-1.81)	0.328
(+)	(-)	3/40	1.99(0.62-6.33)	0.246	1.07(0.33-3.49)	0.905
(+)	(+)	6/54	2.94(1.27-6.82)	0.012	3.02(1.24-7.33)	0.015
Diabetes*Central obesity^†^		121/2561	2.63(1.16-5.98)	0.021	2.67(1.14-6.26)	0.024

Covariates in adjusted model - age, gender, TG, TC, family history of CVD, drinking, smoking, and hypertension. ^†^The interaction term diabetes*central obesity was treated as a variable in the COX proportional hazard model.

Further examination of the additive interaction between diabetes and central obesity on stroke was then executed. Crude and multivariate adjusted measures were calculated. None of the three crude measures of additive interaction between diabetes and central obesity indicated a significant biological interaction (Table 4). After adjustment, significant biological additive interactive effect between diabetes and central obesity on stroke was indicated by the significant AP estimate and its confidence interval, 0.571 (95% CI 0.017-1.125). Approximately 57% of the stroke risk in this cohort can be attributed to the co-effect of diabetes and central obesity.

**Table 4:** **Indexes of additive biological interactive effect of diabetes and central obesity on stroke**

**Measure**	**Unadjusted**			**Adjusted**		
	**Estimate**	**Lower**	**Upper**	**Estimate**	**Lower**	**Upper**
RERI	0.697	-2.578	3.971	1.722	-1.089	4.532
AP	0.237	-0.752	1.225	0.571	0.017	1.125
S	1.558	0.172	14.134	6.878	0.055	853.964

RERI - the relative excess risk because of the interaction; AP - the attributable proportion because of the interaction; S - the synergy index.

## Discussion

Although central obesity has been recognized as a predictor of diabetes [9,10] and diabetes subsequently increases an individual’s stroke risk [12,19], there are very few studies that have examined the interaction of the two risk factors on stroke among general population. This study utilized a prospective cohort study to examine the interactive effect of diabetes and central obesity on incident stroke among Mongolians over the age of 20. We found that the individuals who were both diabetic and centrally obese had significantly higher risk for incident stroke than those who did not have either condition. Furthermore, after adjusting for important confounding factors, individuals with both diabetes and central obesity had a significantly 73% higher risk for stroke than the combination of individuals with either diabetes or central obesity individually. Obviously, there was a significant additive interaction between diabetes and central obesity on stroke among the Mongolians. Approximately 57% of the incident strokes that occurred during the study could be attributed to the interaction of diabetes and centrally obesity. Additionally, the multiplicative interaction between diabetes and central obesity showed a statistically significance. These findings suggest that the risk for stroke in diabetic patients can be moderated and/or reduced by exercise, diet, and other clinical measures if they are also at condition of central obesity.

Diabetes is a well-known risk factor for stroke, a common condition associated with cardiovascular morbidity and mortality [12]. The increased stroke risk present in diabetic individuals can be attributed to a number of factors. Most of which involve metabolic components such as insulin resistance, central obesity, impaired glucose tolerance, and hyperinsulinaemia [12]. Central obesity as defined by waist circumference has been found to be a stronger predictor of diabetes and subsequent stroke risk than overall obesity defined by BMI [5,20-22]. Central obesity participates in the pathway that increases risk for stroke [21]. It leads to an imbalanced production of several metabolic products that potentially affect almost all organ and tissues of the body [22]. Not only is diabetes a major concern in stroke prevention, central obesity is as well. It has been reported that more than 50% of type 2 diabetic patients are centrally obese and possess more risk factors for stroke [23]. The participants with both diabetes and central obesity were also more likely to be current smokers, be hypertensive, and have a family history of CVD in our study. In light of our findings, multiple risk factor managements are essential to prevent incident stroke among the individuals with both diabetes and central obesity.

Among our Mongolian population, the prevalence of diabetes is only 3.67%, much lower than total Chinese (11.6%) [24]. We failed to obtain data on plasma hemoglobin A1c which might lead to an underestimated prevalence of diabetes. The number of individuals with diabetes was limited. As a result, the 95% CI of point estimate of diabetes risk for individuals with both diabetes and central obesity was wide. As well, the estimates of RERI, AP and S varied largely after multivariate adjustment. Although we observed a significant estimate of AP, the 95% CI of AP was very wide. This indicated an unstable result. Even though the multiplicative interaction of diabetes and central obesity on stroke was significant after multivariate adjustment, the interaction still needed further study in other ethnicity populations.

This study has several strengths that deserve mention. To our knowledge, it is the first study to examine the interactive effect of diabetes and central obesity on incident stroke among the Mongolians in China. The study participants were homogeneous regarding their genetic background and environmental exposures, the study data were collected with rigid quality control, and important confounders were measured and controlled in the analysis. In addition, our follow-up time is relatively long and the follow-up rate was relatively high, which enabled us to get a less biased association between exposure variables and outcome events. Despite these strengths a number of limitations should be considered. Because this study specifically observed an Inner Mongolian population, the results may not be readily generalizable to other populations. Another issue influencing generalizability would be the WC cutoffs for central obesity that were arbitrarily set according to previous knowledge and recommendations for Chinese populations. Lastly, although multivariate analysis was conducted, unmeasured confounding factors could still be possible. For instance, participants were not asked to report use of various medications throughout the study period. We also failed to obtain data on dietary and physical activity which contributed to diabetes and stroke risks.

## Conclusions

In summary, this prospective cohort study showed that diabetes and central obesity might synergistically increase an individual’s risk of stroke. We found a significant interaction between diabetes and central obesity on incident stroke in a population of Inner Mongolians. Further research should be done to evaluate the interactive effect of diabetes and central obesity on stroke in other populations. Clinical measures can be taken to help reduce central obesity in diabetic patients. Individuals with both diabetes and central obesity should be intensively followed and treated to prevent incident stroke.

## Abbreviations

TC: Total cholesterol
TG: Triglycerides
LDL-C: Low density lipoprotein cholesterol
HDL-C: High density lipoprotein cholesterol
FPG: Fasting plasma glucose
BMI: Body mass index
WC: Waist circumference
SBP: Systolic blood pressure
DBP: Diastolic blood pressure
CVD: Cardiovascular disease
RERI: The relative excess risk because of the interaction
AP: The attributable proportion because of the interaction
S: The synergy index
HR: Hazard ratio
CI: Confidence intervals
